# Group versus Individualised Minimum Velocity Thresholds in the Prediction of Maximal Strength in Trained Female Athletes

**DOI:** 10.3390/ijerph17217811

**Published:** 2020-10-26

**Authors:** Elias J. G. Caven, Tom J. E. Bryan, Amelia F. Dingley, Benjamin Drury, Amador Garcia-Ramos, Alejandro Perez-Castilla, Jorge Arede, John F. T. Fernandes

**Affiliations:** 1 Higher Education Sport, Hartpury University, Gloucester GL19 3BE, UK; Elias_Caven@hotmail.co.uk (E.J.G.C.); tom.bryan@hartpury.ac.uk (T.J.E.B.); Amelia.Dingley@hartpury.ac.uk (A.F.D.); ben.drury@hartpury.ac.uk (B.D.); 2 Department of Physical Education and Sport, University of Granada, 52005 Granada, Spain; Amagr@ugr.es (A.G.-R.); alexperez@ugr.es (A.P.-C.); 3 Department of Sports Sciences and Physical Conditioning, Faculty of Education, Universidad Católica de la Santísima Concepción, Concepción, Chile; 4 Research Center in Sports Sciences, Health Sciences and Human Development, CIDESD, University of Trás-os-Montes and Alto Douro, 5000-801 Vila Real, Portugal; jorge_arede@hotmail.com

**Keywords:** one-repetition maximum, velocity-based training, squat, bench press, GymAware, agreement

## Abstract

This study examined the accuracy of different velocity-based methods in the prediction of bench press and squat one-repetition maximum (1RM) in female athletes. Seventeen trained females (age 17.8 ± 1.3 years) performed an incremental loading test to 1RM on bench press and squat with the mean velocity being recorded. The 1RM was estimated from the load–velocity relationship using the multiple- (8 loads) and two-point (2 loads) methods and group and individual minimum velocity thresholds (MVT). No significant effect of method, MVT or interaction was observed for the two exercises (*p* > 0.05). For bench press and squat, all prediction methods demonstrated *very large* to *nearly perfect* correlations with respect to the actual 1RM (r range = 0.76 to 0.97). The absolute error (range = 2.1 to 3.8 kg) for bench press demonstrated low errors that were independent of the method and MVT used. For squat, the favorable group MVT errors for the multiple- and two-point methods (absolute error = 7.8 and 9.7 kg, respectively) were greater than the individual MVT errors (absolute error = 4.9 and 6.3 kg, respectively). The 1RM can be accurately predicted from the load–velocity relationship in trained females, with the two-point method offering a quick and less fatiguing alternative to the multiple-point method.

## 1. Introduction

Maximal strength refers to the maximum force or torque that can be exerted by the muscles [1]. Strength is highly important as it is used to prescribe an athlete’s training load [2] and can differentiate between levels of performance within a sport [3]. Moreover, in young athletes, strength is important to maximize athletic performance [4,5]. For example, Comfort and colleagues [4] established moderate to strong relationships between maximal strength and measures of sprint (r > −0.60) and jump (r > 0.76) performance. Thus, it is necessary for practitioners to assess and monitor an athlete’s strength. Maximal dynamic strength is frequently assessed via a one-repetition maximum (1RM) protocol, whereby the maximum load that can be lifted once is identified. However, direct determination of 1RM can be problematic as it is time-intensive, fatiguing and psychologically demanding [6,7]. Though repetitions-to-failure equations are alternatively used because they are time-efficient, they do not negate the fatiguing effects or psychological demand imposed when performing this type of test [6,7]. Fortunately, the recent popularisations of velocity-based devices have enhanced the ability to indirectly determine 1RM [7].

The inverse-linear relationship between movement velocity and load lifted can offer an alternative method to predict 1RM [6,7]. Typically, this relationship is modelled by assessing velocity over multiple submaximal loads (usually from 3 to 8 loads; i.e., the multiple-point method) [7]. Thereafter, 1RM is estimated as the load that is associated with the velocity at 1RM (i.e., the minimum velocity threshold; MVT). Whilst previous work has used an individualised MVT for each individual [8,9], the recent literature suggests that a group-based MVT can predict 1RM with acceptable precision in the bench press [10] and prone bench pull [11]. To our knowledge, only Jukic et al. [9] have compared the precision in the estimation of 1RM between individualised and group-based MVTs and revealed a comparable precision for both MVTs during the deadlift exercise. Like the Weakley et al. [7] commentary, the accuracy of individual versus group MVTs in the prediction of 1RM warrants investigation. Practically, the accurate estimation of 1RM would enhance training through the accurate prescription of the training load. This is even more important for female athletes due to the significant differences in the load–velocity profiles reported in previous studies between men and women [12,13,14].

As the relationship between movement velocity and load lifted is highly linear, it has been proposed that the relationship can be modelled using just two external loads (i.e., the two-point method) [6,15]. For example, Perez-Castilla et al. [6] noted that the multiple-point (i.e., 40, 55, 70 and 85% 1RM) and two-point (i.e., 40 and 85% 1RM) methods provided similar accuracy when predicting 1RM in two upper body pulling exercises. Indeed, a variety of literature confirms that the two-point method can predict 1RM across various different exercises with the same accuracy as the multiple-point method [6,9,15]. Importantly, the two-point method is less time-consuming and induces minimal fatigue when compared to the methods mentioned above (direct determination, repetitions to failure, load–velocity relationship modelled by multiple submaximal loads) [7].

Research on 1RM predictions from the load–velocity relationship in females is lacking. To date, only Perez-Castilla and colleagues [6] reported a comparable precision for men and women in the estimation of 1RM during the lat pulldown and seated cable row exercises. The lack of velocity-based training research with females and sports science in general is problematic for practitioners working with female athletes and likely reflects the patriarchal nature of sports medicine [16,17]. A study which determines the accuracy of 1RM predictions from the load–velocity relationship across resistance-training exercises will aid coaches when prescribing training loads in female athletes. This is particularly important as the slope of the load–velocity relationship is different in males and females [13,14]. Moreover, this would indicate that submaximal loads (i.e., % of 1RM) are associated with different velocities in females compared to males. Therefore, the aim of our study is to determine the accuracy of the load–velocity relationship when predicting the squat and bench press 1RM in trained females. The secondary aim is to determine if the load combination (multiple-point versus two-point methods) and the MVT selected (individualised versus group) affect the accuracy of the 1RM prediction. Given the dearth of comparable studies, we propose the null hypothesis for both of our aims: (1) that there will be no systematic differences between actual and predicted 1RM while their magnitudes would be highly correlated (that is, the accuracy will produce comparable values); and (2) the load combination and the MVT selected will not affect the 1RM prediction accuracy.

## 2. Materials and Methods

### 2.1. Participants

Seventeen resistance-trained females (age 17.8 ± 1.3 years, body mass 69.1 ± 9.6 kg) were recruited via convenience sampling. All participants belonged to the netball team within the sports academy of the host institution and performed bench press (1RM 38.6 ± 7.5 kg) and squat (1RM 86.5 ± 14.7 kg) as part of their training regime (minimum of 1-year systematic resistance training). Participants completed a pre-test health questionnaire and provided informed written consent (and parental consent for those under 18) for the study, which was approved by the host institution’s faculty ethics committee (ETHICS2019-14). The participants were instructed not to consume any ergogenic supplements (for example, caffeine) on the day of testing and to refrain from strenuous exercise in the 3 h before testing. The study adhered to the Declaration of Helsinki.

### 2.2. Experimental Design

The study comprised a randomised crossover design whereby participants attended the strength and conditioning gym on two occasions (one per exercise with 48 h between) during the competitive season. On each occasion (between 10.00 and 12.00 h), participants performed a standardised warm-up before completing an incremental loading procedure until 1RM was attained. The mean velocity (m/s^−1^) of each repetition performed at each load during the incremental loading procedures was recorded using a linear position transducer (GymAware, Kinetic Performance Technology, Canberra, Australia). Thereafter, four different velocity-based methods (2 load combinations × 2 MVTs) were used to predict 1RM. The data of eight loads (~40, 45, 55, 60, 70, 80, 85 and 90% 1RM for bench press and ~20, 30, 45, 55, 65, 75, 85 and 90% 1RM for squat; i.e., the multiple-point method) and only the two distant loads (~40 and 90% 1RM for bench press and 20 and 90% 1RM for squat; i.e., the two-point method) were used for the modelling of the individual load–velocity relationships by applying linear regression models. 1RM was estimated via linear regression equations using the individual load–velocity relationships as the load associated with a group and individual MVT. The group MVT values used to estimated 1RM for bench press and squat was 0.22 and 0.36 m/s^−1^, respectively. For the 1RM prediction from the individual MVTs, each participant’s MVT was used. These ranged from 0.15 to 0.32 and 0.24 to 0.47 m/s^−1^ for bench press and squat, respectively.

### 2.3. Incremental Loading Test

After performing a standardised warm-up, participants performed 1 set of 5 repetitions with a 15 kg free-weight barbell to determine their full range of motion. The incremental loading procedure has been described elsewhere [6]. In brief, participants started with a 15 kg barbell unless their perceived 1RM was > 100 kg, in which case the barbell was loaded to ~20% 1RM. The load was progressively increased in ~10% 1RM increments (~5 to 10 kg) until the mean velocity was < 0.60 m/s^−1^. Thereafter, the load was increased in smaller increments (~1 to 2.5 kg) until participants failed 1 repetition. 1RM was defined as the highest load (kg) that was lifted through a whole range of motion (as determined by the lead researcher). Any repetitions that were not performed with a full range of motion were excluded and repeated. Participants performed 2 repetitions when the mean velocity was > 0.60 m/s^−1^ and 1 repetition when the mean velocity was ≤ 0.60 m/s^−1^. Intra- and inter-set rest was 15 s and 5 min, respectively. The participants received verbal encouragement to improve the accuracy of the 1RM prediction [10]. This procedure was identical for bench press and squat exercise.

### 2.4. Description of Exercises

For the bench press exercise, participants held the barbell with a self-selected width and the pronated grip [18] and lowered it to their chest in a controlled manner and then, without bouncing the barbell, pushed it maximally until full elbow extension. For the squat exercise, with the barbell positioned across their shoulders, participants descended until their hips were below the knee joint and then ascended as rapidly as possible until their knees were at full extension. These exercises were standardised in line with the previous research [19,20,21].

### 2.5. Data Acquisition

GymAware was employed to measure mean velocity from time and displacement. GymAware adopts a variable sampling rate whereby movement is recorded and time-stamped when there is a change of 600 mm (0.0006 m) after which the data are filtered to 50 samples per second. Importantly, this method is deemed a valid and reliable tool in the assessment of mean velocity [21,22].

### 2.6. Statistical Analysis

The data were found to be normally distributed according to the Shapiro–Wilk test (p > 0.05). A two-factor repeated-measures analysis of variance (ANOVA) (MVT [group versus individual] × method [multiple versus two-point]) was applied on the absolute differences between the actual and predicted 1RM separately for each exercise. Furthermore, the validity of the 1RM prediction methods with respect to the actual 1RM was examined through Cohen’s d effect size (ES), the absolute and raw differences (kg) and the heteroscedasticity of the errors (r^2^; relationship of the raw differences between the actual and predicted 1RMs with their average values). Although not indicative of the agreement [21,23], Pearson’s correlation coefficients (r) were calculated to facilitate comparisons to the previous papers. Qualitative interpretations of the r coefficients were defined as follows: *trivial* (0.00–0.09), *small* (0.10–0.29), *moderate* (0.30–0.49), *large* (0.50–0.69), *very large* (0.70–0.89), *nearly perfect* (0.90–0.99), *perfect* (1.00) [24]. The magnitude of the ES was interpreted as follows: *trivial* (<0.20), *small* (0.20–0.59), *moderate* (0.60–1.19), *large* (1.20–2.00) and *very large* (>2.00) [24]. Heteroscedasticity of error was defined as r > 0.32 [25]. Alpha was set at 0.05. All the data were calculated using the SPSS software (version 26, IBM SPSS, INC., Chicago, IL, USA).

## 3. Results

### 3.1. Bench Press

**Figure 1:**
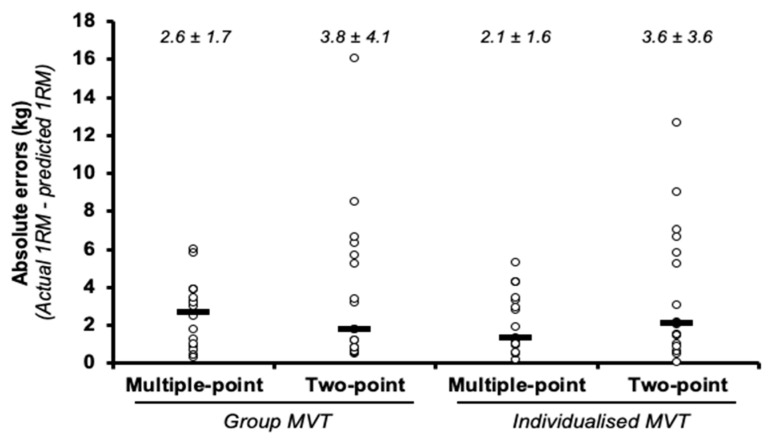
Comparison of the absolute differences (mean ± standard deviation) between the actual one-repetition maximum (1RM) and the 1RM estimated using the different prediction methods in the bench press exercise. Note: the black rectangle denotes the median value, while the circles represent individual data points.

The ANOVA did not reveal a significant main effect for the MVT (F = 0.63, *p* = 0.438) or method (F = 4.3, *p* = 0.054). Furthermore, the MVT × method interaction did not reach statistical significance (F = 0.82, *p* = 0.380). The absolute errors ranged from 2.1 to 3.8 kg (Figure 1, with ESs that were *trivial* and *small* for the multiple- and two-point methods, respectively (Table 1). The correlations ranged from *very large* to *nearly perfect* (r range = 0.84 to 0.97), though higher 1RMs were associated with and increased overestimation (r range = 0.33 to 0.54).

### 3.2. Squat

**Figure 2:**
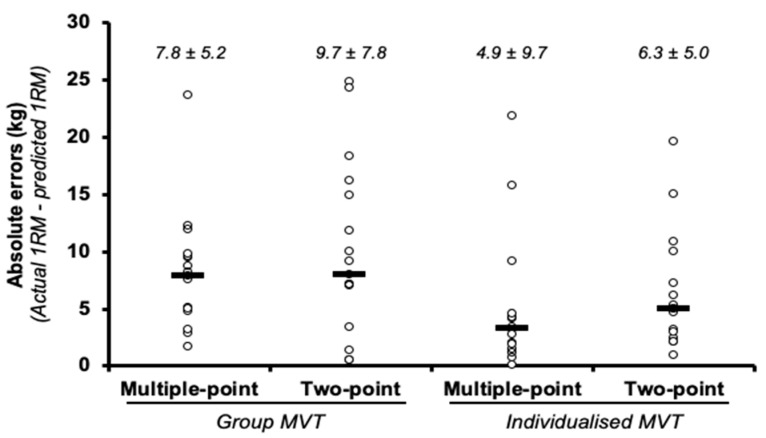
Comparison of the absolute differences (mean ± SD) between the actual 1 repetition maximum (1RM) and the 1RM estimated using the different prediction methods in the squat exercise. Note: the black rectangle denotes the median value, while the circles represent individual data points.

The ANOVA revealed no significant differences for MVT (F = 2.65, *p* = 0.123), method (F = 1.96, *p* = 0.180), or their interaction (F = 0.31, *p* = 0.585). The absolute differences ranged from 4.9 to 9.7 kg (Figure 2) with ESs that were *trivial* and *small* for the multiple- and two-point methods, respectively (Table 2). The correlations for the group methods were *very large* (r = 0.76 and 0.86 for multiple- and two-point methods, respectively) and *nearly perfect* for the individual methods (r = 0.95 and 0.93 for multiple- and two-point methods, respectively). Three of the four prediction methods demonstrated heteroscedasticity of the errors, with higher 1RMs associated with larger errors (i.e., a higher overestimation; r = 0.28 to 0.69).

## 4. Discussion

The aim of the study was to examine the accuracy of the load–velocity relationship when predicting 1RM in trained female athletes. We report here that velocity-based methods can be used to accurately predict bench press and squat 1RM in this population. Our second aim was to determine if the load combination (multiple-point versus two-point) and MVT (individual versus group) affect the accuracy of the prediction. We report that for bench press, both load combinations and MVTs can be used, though practically, a two-point method with a group MVT might be more time-efficient. For the squat exercise, although significant differences were not reached, irrespectively of the load combination, the individual MVT provided 1RM ~ 3 kg more precisely than the group MVT. Holistically, these findings suggest that the load–velocity relationship can be used to predict 1RM in trained female athletes, while there are no evident differences in the precision of different methods and MVTs.

Like previous reports conducted with males, our data demonstrated very large to nearly perfect correlations in the bench press between actual 1RM and predicted 1RMs irrespectively of the methods and MVTs [10,15,26,27,28]. However, measures of association are not indicative of agreement between prediction methods [21]; more important is the absolute agreement and random error between prediction methods. In this study, the small absolute differences and low random errors between actual 1RM and all predictions are in line with previous reports [10,15,26,28,29]. The absolute and random errors in this study, independent of the prediction method, were able to detect the ~ 6 and ~ 8 kg increases in bench press strength that occurred after 8 [30] and 7 [31] weeks of resistance training. For the younger (< 18-year-old) females in our sample, the absolute and random errors, which were similar across load combinations and MVT types, are likely able to detect moderate increases in strength (ES = 0.72) that occur in female youths [32]. It should be noted that all prediction methods for bench press demonstrated small heteroscedasticity, indicating that those with higher 1RMs are subject to greater error (higher overestimation). This is an important consideration, as 1RM is likely to increase with training age in these athletes. Nonetheless, bench press 1RM can be predicted from velocity-based methods in trained female athletes, though using the two-point method with group MVT may be more convenient for practitioners.

Squat demonstrated very large to nearly perfect correlations between direct 1RM and the prediction methods, similar to the previous works [8,33]. The absolute and random errors in this study show favourable agreement and are similar to the previous reports during the squatting exercise [34,35] but in contrast to two reports [8,33]. With the only exception of the two-point method using a group MVT, the remaining velocity-based methods displayed absolute errors lower than 10%. It is important to note that although significant differences were not reached the 1RM predicted using the individual MVTs demonstrated better agreement than the group MVT. The reason for this is unclear but would suggest that the group MVT (0.36 m/s^−1^), did not generally represent the MVT of all the individuals. Indeed, the heterogeneity in the MVTs amongst our sample (from 0.24 to 0.47 m/s^−1^) would support such a suggestion. This might be owing to the squat requiring a multi-jointed coordinated effort to produce the movement. However, the repeatability of the individualised MVT in female athletes is unknown and it is likely to have poor reliability as it has been shown for males [8,11,36]. Therefore, the individualised MVT recorded in one session may be inappropriate when 1RM is estimated in another session. Future studies should compare the precision in the estimation of 1RM between the group and individualised MVTs when the individualised MVT is obtained in a previous session, as it would be the case in real training scenarios (i.e., recording of the individual MVT in one session and use of this individual MVT for 1RM prediction through a training cycle). Regardless of the 1RM prediction method, the errors are all low enough to detect the changes that occur after resistance training in healthy (~ 15 kg [37]) and elite females (~ 12 kg [38]). Furthermore, for the younger participants in this study (i.e., those < 18 years), the errors in this study are likely able to detect moderate (ES = 0.72) improvements in strength that occur in female youths [32]. Like for the bench press exercise, the majority of the errors for squat were heteroscedastic. Therefore, practitioners should be mindful of the larger errors (higher overestimation) in stronger individuals. Collectively, these data suggest that velocity-based methods can be used to predict squat 1RM in trained female athletes. If coaches, however, require greater precision, then the use of individual MVTs is recommended, but it remains to be explored whether this advantage remains when the individual MVT is recorded in a previous session.

Readers should be mindful that we opted not to assess the menstrual cycle in this study. The reasons for this are two-fold. Firstly, the menstrual cycle does not appear to largely affect strength performance [39,40,41,42,43,44,45] or the accuracy in the prediction of 1RM from velocity-based methods [46]. Secondly, our sample was recruited from the sports academy and included U18 athletes. The assessment of the menstrual cycle in youths raises ethical concerns and is not commonplace in the youth literature. However, these potential limitations do provide implications for future research.

## 5. Conclusions

The bench press and squat 1RM can be accurately predicted from the load–velocity relationship in trained female athletes. These data reaffirm previous suggestions in males that 1RM can be predicted with similar accuracy with either the multiple- or the two-point method. From the practical point of view, it is more time-efficient and less fatiguing to use the two-point method. Concerning the type of the MVT used, although acknowledging a generally higher precision of the individual MVT, we failed to show significant differences in the absolute errors between the group and individualised MVTs. However, it is important that future studies elucidate whether the slight differences in favour of the individual MVT still remain when the individual MVT and the load–velocity relationship are obtained in different sessions. Readers should also be aware that low-cost phone applications (e.g., iLOAD, PowerLift), which can accurately measure velocity, are available to use if the most expensive equipment used in our study is not available.

## Figures and Tables

**Table 1:** Differences, associations and heteroscedasticity of the errors between the actual and predicted one-repetition maximums (1RM) during the bench press exercise.

MVT	Method	Predicted 1RM (kg)	Raw Diff (kg)	ES	r*_(Pearson)_*	r*_(Heteroscedasticity)_*
Group	Multiple-point	39.4 ± 8.6	0.8 ± 3.0	0.10	0.94	0.37
	Two-point	41.3 ± 9.0	2.7 ± 4.9	0.32	0.84	0.33
Individualised	Multiple-point	39.5 ± 8.8	0.9 ± 2.5	0.11	0.97	0.54
	Two-point	41.3 ± 9.1	2.8 ± 4.3	0.33	0.89	0.39

Data are mean ± standard deviation. Raw diff, Raw differences; ES, Cohen’s d effect size ([Predicted 1RM − Actual 1RM]/SD both); r*_Peason_*, Pearson’s correlation coefficient; r*_heteroscedasticity_*, heteroscedasticity of the errors.

**Table 2:** Differences, associations and heteroscedasticity of the errors between the actual and predicted one-repetition maximums (1RM) during the squat exercise.

MVT	Method	Predicted 1RM (kg)	Raw Diff (kg)	ES	r*_(Pearson)_*	r*_(heteroscedasticity)_*
Group	Multiple-point	88.6 ± 17.6	3.1 ± 9.1	0.19	0.86	0.37
	Two-point	90.3 ± 17.8	4.7 ± 11.7	0.29	0.76	0.28
Individualised	Multiple-point	88.3 ± 19.5	2.7 ± 7.1	0.16	0.95	0.69
	Two-point	89.4 ± 18.1	3.8 ± 7.2	0.23	0.93	0.49

Data are mean ± standard deviation. Raw diff, Raw differences; ES, Cohen’s d effect size ([Predicted 1RM − Actual 1RM]/SD both); r*_Peason_*, Pearson’s correlation coefficient; r*_heteroscedasticity_*, heteroscedasticity of the errors.
